# Critical Illness Weakness, Polyneuropathy and Myopathy: Diagnosis, treatment, and long-term outcomes

**DOI:** 10.1186/s13054-023-04676-3

**Published:** 2023-11-13

**Authors:** Nicola Latronico, Frank A. Rasulo, Matthias Eikermann, Simone Piva

**Affiliations:** 1 Department of Medical and Surgical Specialties, Radiological Sciences and Public Health, University of Brescia, Brescia, Italy; 2 Department of Emergency, ASST Spedali Civili University Hospital, Piazzale Ospedali Civili, 1, 25123 Brescia, Italy; 3 “Alessandra Bono” Interdepartmental University Research Center On Long-Term Outcome (LOTO) in Critical Illness Survivors, University of Brescia, Brescia, Italy; 4 Department of Anesthesiology, Montefiore Medical Center, Albert Einstein College of Medicine, Bronx, NY USA

**Keywords:** Muscle weakness, Neuropathy, Myopathy, Mobilization, Rehabilitation, Nutrition, Follow-up, Continuum of care

## Abstract

**Background:**

Severe weakness associated with critical illness (CIW) is common. This narrative review summarizes the latest scientific insights and proposes a guide for clinicians to optimize the diagnosis and management of the CIW during the various stages of the disease from the ICU to the community stage.

**Main body:**

CIW arises as diffuse, symmetrical weakness after ICU admission, which is an important differentiating factor from other diseases causing non-symmetrical muscle weakness or paralysis. In patients with adequate cognitive function, CIW can be easily diagnosed at the bedside using manual muscle testing, which should be routinely conducted until ICU discharge. In patients with delirium or coma or those with prolonged, severe weakness, specific neurophysiological investigations and, in selected cases, muscle biopsy are recommended. With these exams, CIW can be differentiated into critical illness polyneuropathy or myopathy, which often coexist. On the general ward, CIW is seen in patients with prolonged previous ICU treatment, or in those developing a new sepsis. Respiratory muscle weakness can cause neuromuscular respiratory failure, which needs prompt recognition and rapid treatment to avoid life-threatening situations. Active rehabilitation should be reassessed and tailored to the new patient’s condition to reduce the risk of disease progression. CIW is associated with long-term physical, cognitive and mental impairments, which emphasizes the need for a multidisciplinary model of care. Follow-up clinics for patients surviving critical illness may serve this purpose by providing direct clinical support to patients, managing referrals to other specialists and general practitioners, and serving as a platform for research to describe the natural history of post-intensive care syndrome and to identify new therapeutic interventions. This surveillance should include an assessment of the activities of daily living, mood, and functional mobility. Finally, nutritional status should be longitudinally assessed in all ICU survivors and incorporated into a patient-centered nutritional approach guided by a dietician.

**Conclusions:**

Early ICU mobilization combined with the best evidence-based ICU practices can effectively reduce short-term weakness. Multi-professional collaborations are needed to guarantee a multi-dimensional evaluation and unitary community care programs for survivors of critical illnesses.

## Introduction

In 1984, Bolton described the first 5 cases of “critically ill polyneuropathy” in patients with “adult” respiratory distress syndrome (as these syndromes were called at that time) [1]. Initially, the condition described seemed very rare, as it took 4 years to describe these 5 patients, whose clinical presentation was characterized by profound weakness of “neck, chest wall, abdomen and limb muscles” and absent deep tendon reflexes. In 1987, in a following study of 17 patients, critical illness polyneuropathy (CIP) was associated with “failure to wean” the patient from the ventilator, a diagnosis that was made when “despite partial resolution of the critical illness, the patient could not tolerate a reduction in the frequency of mandatory mechanical ventilation” [2].

In 1985 [3] and then in 1991 [4], Op de Coul described 22 patients with combined CIP and critical illness myopathy (CIM) and first proposed the term polyneuromyopathy. In 1995, Leijten found that ICU patients diagnosed with CIP had persisting motor deficits at 1 year, indicating the need for long-term follow-up of ICU survivors [5]. In 1996, we found that CIP may present as an isolated electrophysiological alteration in the early stage of disease (i.e. with maintained nerve structural integrity at nerve biopsy), whereas axonal degeneration occurred typically in patients with persisting symptoms [6]. We described these findings using the concept of bioenergetic failure [7], where combined CIP and CIM can cause reversible tetraplegia in critically ill comatose patients. During the last 25 years, we learned that combined CIM and CIP is the most common manifestation of acute neuromuscular weakness in the ICU [8, 9].

The term “ICU-acquired weakness” (ICUAW) appeared in 1993 in a report of 5 patients with acute necrotizing myopathy [10]. However, it was not until 2002 that the “ICU-acquired paresis” was systematically investigated in a multicenter French study using the Medical Research Council sum score (MRCss) in patients awakening after prolonged sedation [11]. The investigators found that 25% of patients developed a global, flaccid weakness. A similar incidence of ICUAW of around 25%, was found in 2008 in an American study that used both the MRCss and handgrip dynamometry for assessing muscle strength [12]. Sensitive diagnostic tools have shown that the incidence of ICUAW in patients who slowly recover from the critical illness is 43% (interquartile range 25%-75%) [13] and is associated with prolonged mechanical ventilation and ICU stay [13], increased ICU, hospital, 1-year [14] and 5-year mortality [15], in-hospital costs [16], and impaired long-term physical function [15] and aerobic capacities [17]. With an estimated 13–20 million patients admitted to the ICU worldwide, more than 6 million new patients will experience ICUAW annually [18]: an apparently rare condition has turned into a common and serious ICU complication with long-term consequences.

This article is meant to guide clinicians caring for adult critically ill patients in diagnosing severe muscle weakness associated with critical illness at various stages during the hospital stay and after hospital discharge. We also propose strategies on how to approach muscle weakness during the various stages of the patient’s difficult journey from ICU admission back into the community. In proposing this unitary vision, we emphasize that severe weakness may not be solely “acquired” in the ICU. Instead, it should be seen as the “extreme end of a spectrum of weakness that begins with any serious illness regardless of care location” [19]. Therefore, in this review we will use the term Critical Illness Weakness (CIW), in assonance with CIP and CIM, as a more appropriate and specific description of the generalized weakness that affects acutely ill patients.

### In the ICU

CIW presents as a symmetrical muscle weakness arising *after* the onset of a critical illness, affecting the limbs and the respiratory muscles, and sparing the facial muscles [19, 20]. There is no agreement as to whether diaphragm weakness (DW) should be considered an integral part of the syndrome or a separate entity. Historically, the coexistence of limb weakness and DW was considered the typical presentation of the syndrome [1]. However, DW is poorly correlated with CIW [21–23], suggesting that CIW and DW might be distinct entities. The concept of respiratory muscle weakness has further expanded in recent years to involve extra-diaphragmatic inspiratory muscles and expiratory muscles weakness, in addition to DW [24–26]. Respiratory muscle weakness is associated with weaning failure, longer duration of mechanical ventilation, and, if present at ICU discharge, increased risk of in-hospital mortality, ICU and hospital readmission [21] and worse physical function and quality of life at long-term evaluation [27].

CIW is a diagnosis of exclusion when generalized and symmetrical limb weakness after ICU admission develops in the absence of other etiology [20]. These features are important to distinguish CIW from Guillain-Barré syndrome, myasthenia gravis, acute myopathies and neuropathies, amyotrophic lateral sclerosis, drug overdose, envenomation and other conditions that may cause acute neuromuscular respiratory failure [28, 29]. Differential diagnosis is usually obvious, as these conditions are typically a cause of ICU admission, and not a complication of critical illness during ICU stay [29]. Conversely, CIW is commonly diagnosed in patients with persisting critical illness [30] who have normal muscle strength at ICU admission: among patients still in the ICU on day 8 after admission, the incidence of CIW is higher than 50% [16]. Some neuromuscular diseases may have an acute onset, and some chronic conditions may have sudden exacerbations, thereby presenting with acute respiratory failure [28]. These cases represent a diagnostic challenge, because limb weakness becomes apparent when the acute phase of respiratory failure has resolved and patients are weaned off sedatives, thus posing the problem of whether the weakness is pre-existing upon admission to the ICU or developed during the ICU stay. If the etiological diagnosis is not clear at ICU admission, differential diagnosis requires meticulous attention to neurological signs [31] (i.e. weakness of eye muscles with ptosis and ocular palsy in myasthenia gravis; asymmetrical distribution of muscle weakness such as hemiparesis in acute ischemic stroke; poorly reactive dilated pupils as in botulin intoxication; autonomic signs, such as cardiac arrhythmias or blood pressure fluctuations as in Guillain-Barré syndrome) and specialized neurophysiological investigations [29, 32].

**Fig. 1:**
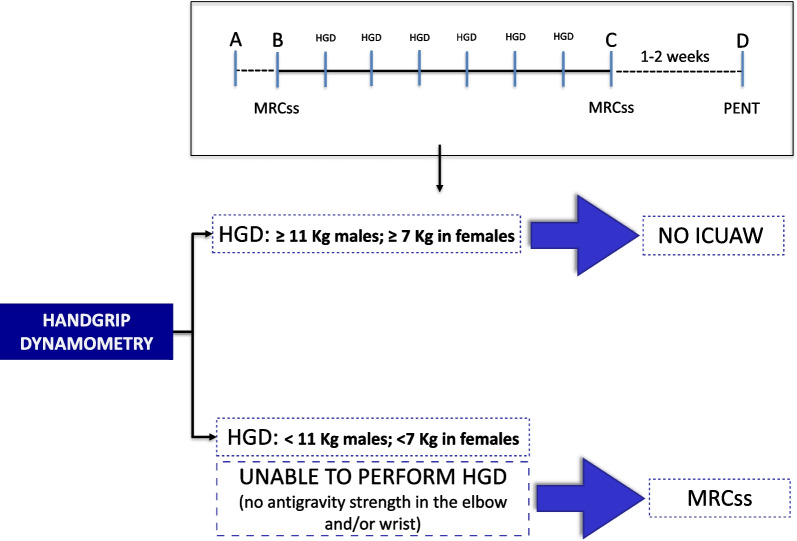
Two-tier muscle strength assessment (2T-MSA) : MRC sum score (MRCss) should be assessed as soon as the patient awakens and at ICU discharge. Handgrip dynamometry (HGD) can be used during the ICU stay as a quick screening tool. **A**: ICU admission; **B**: patient cooperative; **C**: ICU discharge. **D**: persisting weakness. CIW: critical illness weakness. PENT: peroneal nerve test. Adapted from Parry SM, et al. [33]

In cooperative patients, muscle strength should be assessed at the bedside using the MRC scale, handgrip dynamometry (HGD), or both [19]. MRC sum score (MRCss) should be assessed as soon as the patient awakens and at ICU discharge at the very minimum, while handgrip dynamometry can be used as a quick screening tool throughout the ICU stay (Fig. 1) [33]. Critical care nurses may also identify signs and symptoms of severe weakness using a subjective assessment tool, and their findings should also be considered during interprofessional rounds [34].

**Fig. 2:**
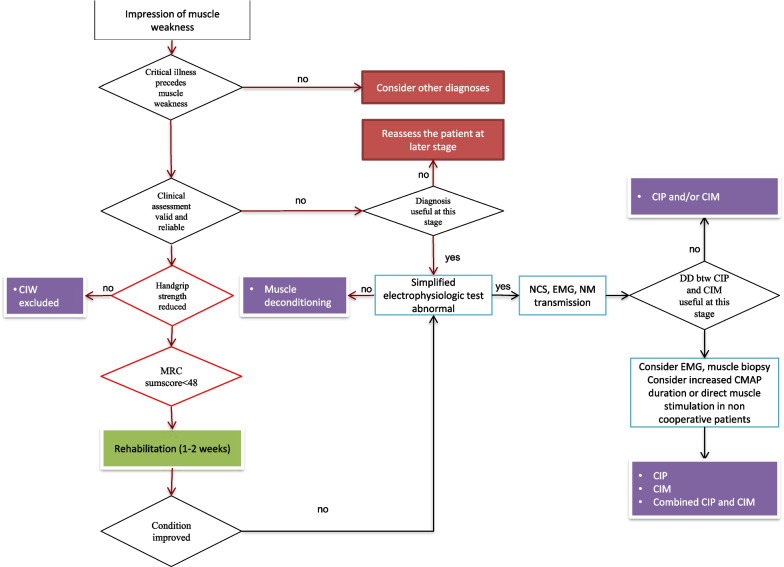
Diagnostic algorithm for critical illness weakness (CIW). DD = differential diagnosis. CIM = critical illness myopathy. CIP = critical illness polyneuropathy. CMAP = compound muscle action potential. EMG = electromyography. MRC = Medical Research Council. NCS = nerve conduction study. NM = neuromuscular. Cut off handgrip strength values are below 7 kg for female and below 11 kg for males. For information on increased CMAP duration and direct muscle stimulation see Latronico N, Bolton CF [8]. Adapted from Latronico N, Gosselink R [35]

The term CIW condenses various neurophysiological and neuropathological entities with different prognoses into one single clinical entity. Therefore, if weakness persists, electrophysiological investigations of peripheral nerves and muscles and, in selected cases, muscle biopsy (Fig. 2) will eventually be required to define the specific pathological diagnosis and tailor the treatment plan [35]. In the era of “precision critical care medicine” [36], a more specific diagnosis may inform long-term prognosis (Table 1) [8] and may hopefully enable targeted pharmacological treatments in the near future, as it is for other neuromuscular disorders with axonal nerve transport dysfunction [37] or muscle electrical excitability alterations.

**Table 1:** Generalized neuromuscular conditions associated with critical illness. Adapted from Latronico N, Bolton C.F.[8]

Condition	Incidence	Clinical features	Electrophysiologic findings	Serum creatine kinase	Muscle biopsy	Prognosis
POLYNEUROPATHY Critical illness polyneuropathy	Common	Flaccid limbs; respiratory weakness	Axonal degeneration of motor and sensory fibres	Nearly normal	Denervation atrophy	Variable
NEUROMUSCULAR TRANSMISSION DEFECT Transient neuromuscular blockade	Common with neuromuscular blocking agents	Flaccid limbs; respiratory weakness	Abnormal repetitive nerve stimulation studies	Normal	Normal	Good
CRITICAL ILLNESS MYOPATHY Thick-filament myopathy	Common with steroids, neuromuscular blocking agents, and sepsis	Flaccid limbs; respiratory weakness	Abnormal spontaneous activity	Mildly elevated	Loss of thick (myosin) filaments	Good
Acute myopathy with scattered necrosis	Common	Flaccid limbs; respiratory weakness	Myopathy	Mildly or moderately elevated	Scattered necrosis	Variable
Acute myopathy with diffuse necrosis (necrotising myopathy of intensive care)	Rare	Flaccid weakness; myoglobinuria	Severe myopathy	Markedly elevated, myoglobinuria	Marked necrosis	Poor
Disuse (cachectic) myopathy	Common	Muscle wasting	Normal	Normal	Normal or type II fibre atrophy	Variable
RHABDOMYOLYSIS	Rare	Flaccid limbs	Near normal	Markedly elevated (myoglobinuria)	Normal or mild necrosis	Good
COMBINED POLYNEUROPATHY AND MYOPATHY	Common	Flaccid limbs; respiratory weakness	Indicate combined polyneuropathy and myopathy	Variable	Denervation atrophy and myopathy	Variable

In patients with impaired brain function who are unable to engage with voluntary muscle strength measurements, instrumental diagnosis may help to establish a diagnosis of CIP or CIM. The simplified electrophysiological peroneal nerve test (PENT) can quickly detect a reduction of the compound muscle action potential amplitude [38] and may indicate the need for further neurophysiological investigations (Fig. 2) [32]). In patients with septic shock requiring moderate or deep sedation, the use of PENT allows early diagnosis of CIP and CIM, before muscle weakness becomes clinically evident [39]. Isolated electrophysiological alterations of peripheral nerves and muscles (i.e. without discernible muscle weakness) at ICU discharge can differentiate pre-hospital discharge functional status [40] and are independently associated with increased 5-year mortality [15]. Neuromuscular ultrasound (NMUS) shows promise as an easily repeatable, noninvasive technique for detecting CIP and CIM. In CIP, NMUS may describe changes in thickness, cross-sectional area and echogenicity in nerves of the upper and lower extremities [41]. Increased nerve cross-sectional area, suggesting nerve enlargement, may distinguish between CIP and CIM and correlates with the duration of mechanical ventilation and days spent in the ICU [41]. In CIM, NMUS may identify changes in limb muscle mass and architecture by measuring the muscle layer thickness, cross-sectional area and echointensity, which correlate with functional outcomes and mortality [42–44]. NMUS can identify necrosis of muscle fibers [45], which has a worse prognosis than loss of thick myosin filaments [8], and fascial inflammation and fasciitis, which may cause severe pain [19, 45]. Ultrasound can be used to assess the diaphragm thickening fraction which predicts ventilator weaning failure [21], to titrate ventilatory support, and to evaluate patient-ventilator interaction, diaphragmatic paralysis and progression of atrophy in mechanically ventilated patients [19, 46]. Other more contemporary techniques include force measurements by combining electrical or magnetic supramaximal stimulation applied either over a motor nerve or a muscle belly with an ergometer. Some ergometers allow the measurement of evoked muscle force in response to stimulation on three muscle groups: adductor pollicis, ankle dorsiflexors, and quadriceps. These techniques have been recently reviewed in detail [47].

CIW and delirium are common ICU complications and often coexist [19, 48]; indeed, “neck down” neuromuscular disorders are often associated with “neck up” brain disorders [49] (see Fig. 2 in [19]). CIW cannot be reliably assessed if the patient is delirious; even hyperactive delirium can be difficult to assess if the patient is severely weak. Since the facial muscles are unaffected in CIW, the only sign of agitation can be a ceaseless crescendo and diminuendo of lateral head movements. In patients with CIW who also have delirium, sternal or nail bed pressure will lead to facial grimacing despite reduced or absent limb movement [8].

In comatose patients, the absence of reflex motor response of the limbs to pain caused by CIP and CIM can be reversible [6]. This can be seen in non-neurological ICU septic patients with encephalopathy [50] or diabetic patients with hyperosmolar coma [51], but also in neurologic patients developing sepsis [52]. In these cases, brainstem reflexes, such as pupillary light and corneal reflexes, remain intact [6, 53]. By contrast, the same clinical picture portends a grim prognosis if it is caused by an acute brain disease and is associated with altered brainstem reflexes [31]. Thus, a careful neurologic examination is vital to differentiate whether the absent motor responsiveness is due to peripheral or central causes. Since predicted outcomes can affect patient’s treatment [54], an accurate differential diagnosis is also important to avoid a decision to withdraw life-sustaining therapy in patients with reversible paralysis [55].

**Fig. 3:**
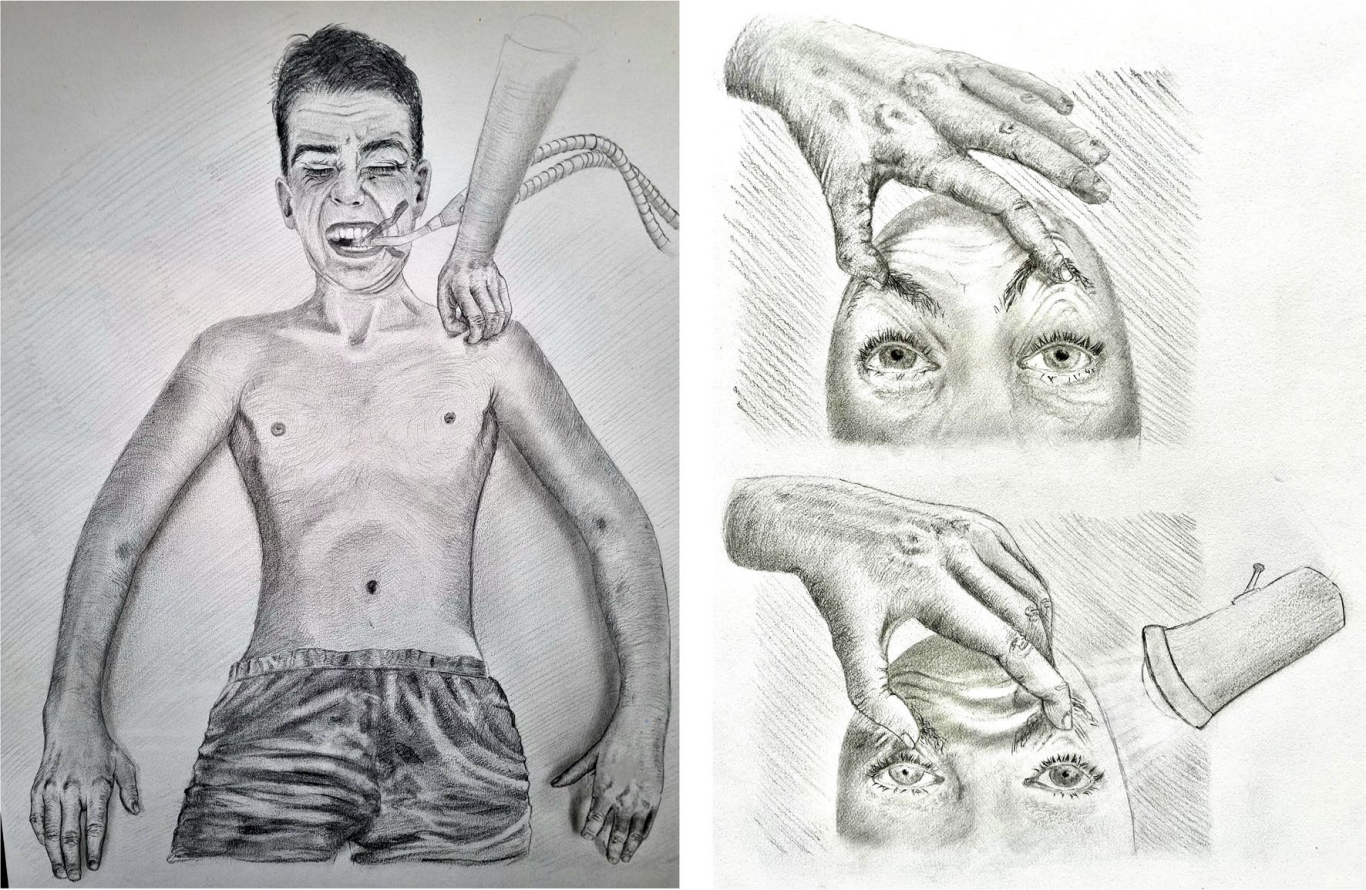
Facial grimacing with absent limb motor responsiveness after painful stimulation in a patient emerging from coma (**a**). Bilateral pupillary light reflex is maintained (**b**)

In patients emerging from coma, the presence of facial muscle movements in response to pain (i.e. grimacing) combined with normal brainstem reflexes, nystagmus and slowly roving, spontaneous, conjugate eye movements suggests a peripheral mechanism of severe muscle weakness (Fig. 3) [53].

In patients with cognitive-motor dissociation (covert consciousness) [56, 57], who recover their cognitive function with a substantially delayed recovery of muscle function, electrophysiological neuromuscular investigations are paramount to consolidate or exclude the diagnosis of CIP and CIM as the cause of motor impairment [58].

What should be done once CIW is documented in the ICU? Physical rehabilitation is important and should be started soon after CIW is diagnosed. Functional exercises, such as sitting, standing, and walking, are the most effective rehabilitation goals in the ICU [59] and should be reevaluated continuously as the patient’s condition changes [60]. Since bed resting is an important risk factor for CIW [61], preventive strategies such as early mobilization, started when the patient condition is physiologically stable (usually within 2–3 days of ICU admission and hence, before the development of CIW), is recommended by clinical practice guidelines [62]. Early mobilization can mitigate the physical dysfunction at hospital discharge, provided that patient safety is guaranteed and the level and duration of activity are tuned to individual needs [59, 63, 64]. Device-assisted physical therapy is increasingly used to facilitate early mobilization even though guidelines do not yet recommend its use [65]. The ABCDEF bundle (Assess, prevent, and manage pain; Both spontaneous awakening and breathing trials: Choice of Analgesia and Sedation; Delirium assess, prevent, and manage; Early Mobility and Exercise; Family engagement/empowerment), one of whose elements is early mobilization, enables a holistic integrated approach to the patient [66]. Unwanted deep sedation prevents patient interaction with the ICU team and the family, is a risk factor for delirium and causes immobility, contributing to long-lasting physical and cognitive impairments. A protocol of light or no-sedation is acceptable to most patients [67], is perfectly suited for early mobilization and occupational therapy, may reduce delirium duration and improve long-term weakness and cognitive outcomes [68].

### In the hospital ward

Ideally, no patient should be discharged from the ICU without having the muscle strength assessed and the MRCss recorded, as even a mild MRCss reduction ≤ 55 is associated with increased long-term morbidity and mortality up to 5 years [15, 69]. Diagnosis of CIW should be reported in the clinical chart and the need for discharge to a rehabilitation center should be anticipated. However, CIW remains an underrecognized disease [70, 71], and many patients are transferred to the general ward without proper assessment of muscle strength. This may expose the patient to serious complications on the floor, such as acute neuromuscular respiratory failure, a life-threatening condition with a difficult diagnosis and insidious progression. Weak cough with an inability to clear secretions, cough after swallowing (indicating dysphagia), staccato speech (i.e. the need to pause between words while speaking), rapid shallow breathing, and breathlessness when swallowing are important warning signs indicating imminent respiratory failure [29, 72, 73]. Arterial blood gases worsen only late in the course of the disease and chest X-ray is often normal or shows only reduced chest expansion [29, 72]. If CIW is diagnosed, patients should receive immediate attention; sepsis and respiratory muscle weakness should be actively sought, and, if present, should dictate close patient monitoring, or even ICU readmission if this is in the best interest of the patient. Dysphagia, an additional neuromuscular dysfunction [74] which is common after tracheal intubation and mechanical ventilation [75], is worth being evaluated because early bedside assessment [76] can reduce serious complications such as choking, aspiration pneumonia, and respiratory failure [77].

Severe weakness may not be solely “acquired” in the ICU. In patients with sepsis, which is a major problem in hospital wards where in fact 50% of patients with sepsis are treated [78, 79], CIW develops in up to 64% of patients [13]. The incidence of CIW may be similar in patients with sepsis admitted to internal medicine wards, where in fact the "Sepsis-3" diagnostic criteria work just as well as in the ICU and the sequential organ failure assessment (SOFA) score is closely associated with in-hospital mortality [79]. This suggests that the process leading to the failure of body organ systems, including that of the neuromuscular system, is the same regardless of the environment in which sepsis develops. Sir William Osler describing sepsis in 1892, well before the intensive care medicine was born, observed: “The constitutional disturbance is marked and there are loss of appetite, nausea and vomiting, and, as the disease progresses, rapid loss of flesh” [80]. To date, CIW outside the ICU is described only anecdotally [81] or in small series [72], and this is an important area for future clinical investigations. Acute kidney injury (AKI) and days on renal replacement therapy are risk factors for CIW [82]. AKI alters the tissue use of amino acids even in its early stage, thereby making it plausible that AKI exacerbates CIM [83]. Interestingly, CIP has long been described outside the ICU in patients developing sepsis in the course of severe kidney failure [84]. Patients with abdominal infections, pancreatitis and trauma admitted to surgical wards are also prone to develop CIW [81]. Therefore, CIW can be a common encounter for general ward physicians and nurses or critical care outreach teams. Assessing muscle strength should become a common practice in these patients, as is measuring the blood pressure or assessing pain, and the term CIW can help break down the myth that severe weakness is observed only in ICU patients. Provision of longitudinal follow-up can facilitate maintaining active rehabilitation initiated in the ICU without unwanted interruptions [85]. In the UK, a Post-ICU Presentation Screen (PICUPS) has been developed as a screening tool to identify rehabilitation needs in patients stepping down from ICU and the general ward into rehabilitation and the community [86].

As suggested by convenors in a round table meeting that was held in Brussels in March 2016, we should “embrace the continuum of illness including pre-ICU trajectory, recovery, and adaptation” to optimize outcomes [19]. Post-ICU discharge is an integral part of this continuum, and, as such, it needs careful planning and a coordinated approach by multidisciplinary teams tailoring management to the actual patient’s needs [87]. With “byzantine” post ICU-transitions [87], less than 1 in 10 patients recover to functional independence.

### After hospital discharge

When sorrows come, they come not single spies, but in battalions (Shakespeare, Hamlet, Act IV, Scene V).

**Fig. 4:**
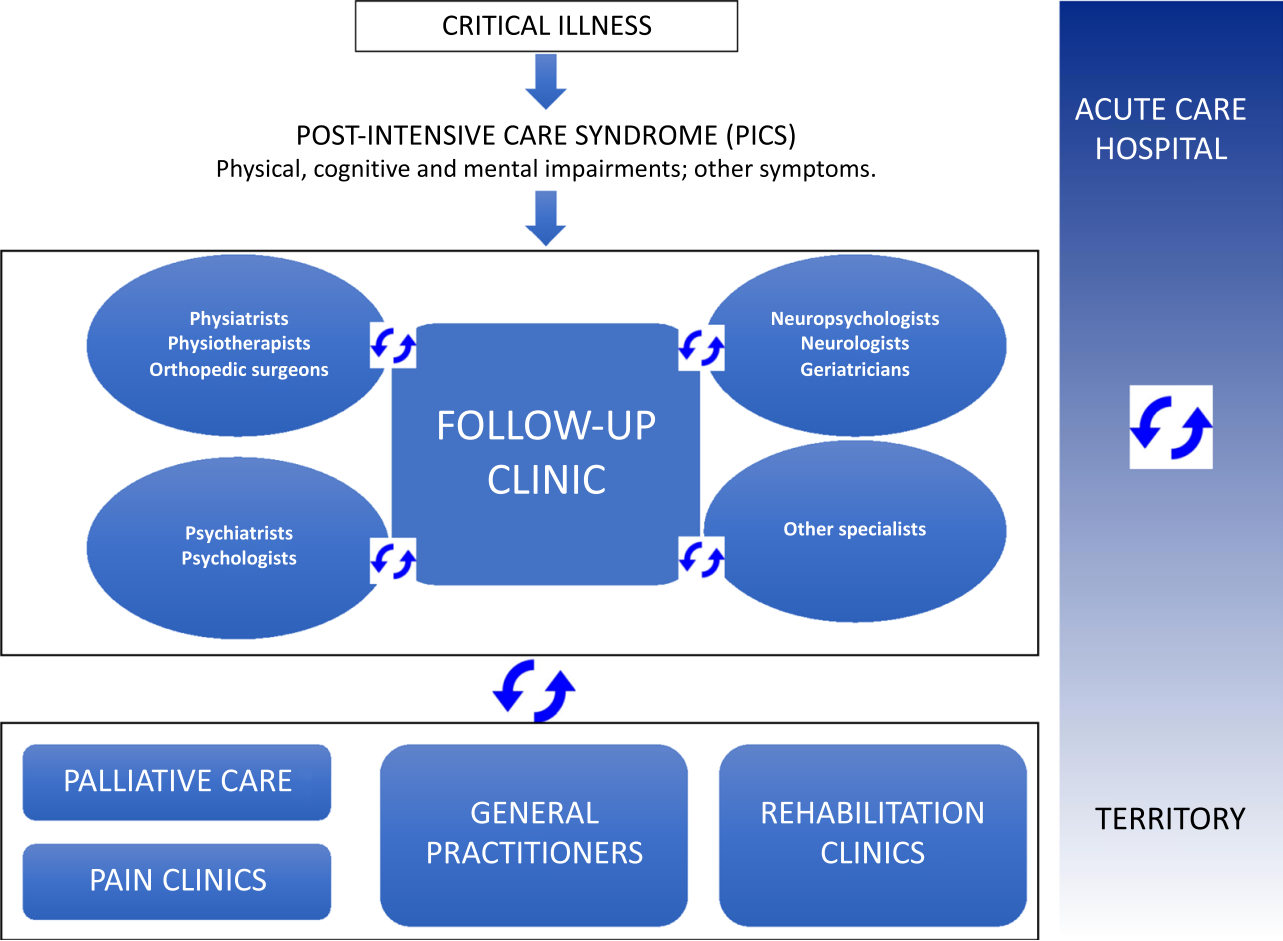
Interactions of the Follow-Up Clinic with health care specialists and structures inside and outside the acute care hospital. Adapted from Latronico N, Piva S, Rasulo FA [95]

Survivorship is a crucial challenge facing modern critical care medicine, as Iwashyna predicted more than 10 years ago [88]. Determinants of long-term physical impairment are myriad [89] and include pre-existing conditions as well as ICU and post-ICU factors. Importantly, ICU patients develop new pathological processes that are “both organic and iatrogenic” [49]. Meticulous attention should be paid to reducing iatrogenic events, such as deep sedation or immobility, and there must be a continuum of care for patients—and families too—extending from the ICU to community or primary care to provide optimal outcomes for critically ill patients [87]. In the UK, follow-up programs after ICU admission are embedded in national rehabilitation guidelines [85]. Physical disability after acute care hospital discharge does not come in isolation; instead, it is often associated with cognitive and mental impairments [87], which all together impact the quality of life and return to work (*Case Vignette*). Therefore, the approach to ICU survivors should consider a multidisciplinary model of care, as recently emphasized also in COVID-19 survivors [90]. This is reinforced by the fact that, with improving experience, other conditions have been described to be part of post-intensive care syndrome (PICS), such as new-onset diabetes (particularly in patients with stress hyperglycemia during the ICU stay [91]), endocrine disturbances, sleep disorders, dysphagia, sexual dysfunctions [92, 93], pain and autonomic dysfunction [19, 94]. Follow-up clinics for patients surviving critical illness may serve this purpose by providing direct clinical support to patients, managing referrals to other specialists and general practitioners, and serving as a platform for research on the natural history of PICS and the assessment of new therapeutic interventions (Fig. 4) [90, 95]. Follow-up clinics may become an important referral for family physicians, since ICU survivors consult them more frequently than non-ICU patients with similar morbidities [96]; therefore, it is essential that family physicians are aware of the close link between acute ICU events and long-term disability (Case Vignette).

### Case vignette

A previously healthy 57-year-old woman was referred by her family physician to the follow-up center 11 months after her ICU admission for pneumonia and septic shock. In the ICU, she was sedated with propofol, received neuromuscular blocking agents for mechanical ventilation and was treated with antibiotics, vasopressors, steroids, and opioids for pain control and suffered hypo-active delirium for 4 days. After 11 days, the patient was extubated and two days later was transferred to the division of pulmonary medicine. Although she was unable to lift her arms and legs against gravity, a formal evaluation of muscle strength was not done. She was discharged home on day 26. Two months later, she resumed her job as a bank accountant but quit after a few weeks because she felt fatigued, and her mind was foggy. She also reported difficulty walking and climbing stairs. During the 11 months after discharge, she was seen several times by her family physician and by specialists including a pulmonologist, a neurologist, a cardiologist, a psychologist, and an expert in non-conventional medicine. A treatment with anti-depressants was temporarily beneficial.

The family physician, after attending a conference on post-intensive care syndrome, prescribed a visit to our follow-up clinic, where the patient demonstrated signs of a sensorimotor neuropathy with reduced muscle strength especially in the lower limbs and gait disturbances. Electromyographic findings were consistent with a sensory-motor axonal polyneuropathy. The 6-minute walk test and handgrip dynamometry were 70% and 78% of percentage predicted, respectively. The Montreal Cognitive Assessment (MOCA) was 25, indicating mild cognitive impairment. The mini-nutritional test score was 10 indicating risk for malnutrition. The Hospital Anxiety and Depression Scale (HADS) for depression showed borderline depression (score: 9), whereas HADS for anxiety showed severe anxiety (score: 13). There was no evidence of post-traumatic stress disorder. The Fatigue Severity Score was 23, indicating severe fatigue. The physical and mental component summary scores of the SF36 were also altered (scores: 42 and 44, respectively).

The patient was informed that all her symptoms were part of the post-intensive care syndrome and that she could improve with time. She was prescribed a physical therapy program with a physiotherapist every weekday, consisting of walking at least 30 minutes at a brisk pace twice a day every day, and a diet with 35Kcal/Kg and amino acids supplement (5 to 10 mg daily). She was also referred to a clinical psychologist.

The patient was very pleased with such a comprehensive examination and was very relieved that the condition was potentially reversible, because she was convinced that she had a progressive degenerative disease or cancer.

At two years, when last seen, the patient’s condition had greatly improved in all PICS domains. She had returned to work, albeit with reduced hours, and enjoyed renewed activity in social life.

Severe muscle weakness may persist for months to years after hospital discharge, and many patients may not recover to their pre-ICU condition. In the ICAP study in survivors of acute lung injury, CIW was diagnosed in 36% of patients at hospital discharge and was still persisting in 14% at 1 year and 9% at 2 years [97]. In the ALTOS study in ARDS survivors, 6% of patients had persisting CIW at 1 year [98]. Physical sequelae are also common in sepsis survivors; indeed, hundreds of thousands of sepsis survivors, not only patients treated in the ICUs [99], face the challenge of persisting physical disability [100]. Sepsis survivors often report a significant deterioration of their perceived physical function compared with pre-admission status [101]. When physical function is measured using objective methods in older patients, sepsis negatively impacts activities of daily living (ADL) and instrumental ADL (IADL), particularly in patients with better baseline physical functioning [102]. In surgical patients with chronic critical illness (≥ 14 ICU days), sepsis has a devasting effect on physical function: patients are not only limited in their ADL, but they can also be confined to bed or a chair most of the day for months after ICU discharge [103]. Physical impairments, including reduced 6 min walk distance (6MWD), 4 m gait speed, hand grip, MRCss and pulmonary function, are equally common in patients with hyper-inflammatory and hypo-inflammatory sepsis-associated ARDS subphenotypes [104], suggesting that inflammation may not be a key driving mechanism of long-term functional impairments in this population.

Recovery of physical functioning requires not only the recovery of muscle strength. Accordingly, the physical assessment in ICU survivors should consider a comprehensive approach that evaluates factors at the level of the body function and structure, and the whole person in a standardized environment and in a societal context (Table 2) [105–107]. As an example, reduced 6MWD indicates a whole person’s physical dysfunction, since the test evaluates the integrated responses of the neuromuscular units, muscle metabolism, pulmonary and cardiovascular systems, and blood, but also the joints (range of motion), the brain (proprioception, balance, cognition), and the mind (motivation) [95].

**Table 2:** Health domains of the post-intensive care syndrome, measured outcomes, and instruments.

Domains of Post-Intensive Care Syndrome	Measured Outcome	Instruments
Physical domain		
Body level		
Function	Muscle weakness	MRC scale, handgrip or handheld dynamometry
	Critical illness neuromyopathy	Electromyography
	Fatigue	FSS, FACIT-F
Structure	Myopathy	Muscle biopsy
Whole body in a standardized environment: Activities	Performance-based activity limitations	Six-minute walk test, timed up-and-go
	Self-reported activity limitations	SF-36 physical functioning
Whole body in in a social context: Participation	Participation restrictions	ADL, IADL, return to work, SF-36 role physical
Cognitive domain		
	Subjectively reported cognitive impairment	Cognitive outcomes reported by patients or their caregivers
	Objectively reported cognitive impairment: screening tests	MoCA
	Objectively reported cognitive impairment: batteries of cognitive tests	RBANS
Mental health domain		
	Depression	HADS-depression
	Anxiety disorders	HADS-anxiety
	PTSD	PTSD checklist for DSM-5, IES

Adapted from Latronico N, et al. [95]

The list of instruments includes some examples, it is not intended to provide a comprehensive list of all instruments available

MRC, Medical Research Council. EMG, electromyography. FSS, fatigue severity score. FACIT-F, Functional Assessment of Chronic Illness Therapy-Fatigue Scale. SF-36, 36-Item Short-Form Health Survey. ADL, activities of daily living. IADL, instrumental activities of daily living. MoCA, Montreal Cognitive Assessment. RBANS, Repeatable Battery for the Assessment of Neuropsychological Status. HADS, Hospital Anxiety and Depression Scale. PTDS, Posttraumatic Stress Disorder Checklist for Diagnostic and Statistical Manual of Mental Disorders-5 (DSM-5). IES, Impact of Event Scale

Chronic muscle denervation consistent with previous CIP can be found up to 5 years after ICU discharge in > 90% of long-ICU stay patients (> 28 days) [108]. CIP persists longer than CIM and causes prolonged muscle weakness and paralysis [109]. Even in milder cases with complete functional recovery, CIP can cause reduced or absent deep tendon reflexes, stocking and glove sensory loss, and painful hyperesthesia [110]. The PENT can be usefully implemented to define the cause(s) of symptoms, as it is easily acquired, is quick (10 min [38]) and can be used to screen patients needing more in-depth electrophysiological investigations, replicating the scheme used during the ICU stay (Fig. 2). 

In the general adult population in the USA, peripheral neuropathy with decreased lower-extremity sensation commonly affects adults with diabetes but is also described in subjects with normoglycemia and is independently associated with mortality [111]. CIP is itself a peripheral neuropathy involving the sensory nerves, not only the motor nerves, and is associated with stocking and glove sensory loss and painful hyperesthesia in ICU survivors [73]. As such, CIP might contribute to the problem of decreased sensation in the lower limb and increased mortality described in the general adult population. This further emphasizes the need for actively seeking CIP not only in ICU survivors with persistent weakness, but also in those with persisting altered sensation in lower extremities. If CIP is diagnosed, referral to a rehabilitation physician and physiotherapist is warranted [112], particularly for patients with high comorbid burden [113].

Optimal nutrition also needs to be addressed. Nutritional status is seldom considered in the context of ICU recovery, even though critical illness is associated with malnutrition [114]. Body weight loss, a cardinal sign of malnutrition, is common in ICU patients who lose nearly 2% of lean body mass per day during the first week of their ICU stay [115]. At 1 year, only one-third of patients have regained their body weight [116] and, among those who gain weight, there can be an increase in fat mass rather than lean body mass [115]. Reasons for persisting malnutrition include physiological, functional, psychological and organizational barriers and have been reviewed elsewhere [115]. The nutritional status can be examined using simple tools, such as the mini-nutritional assessment short-form [117]. Factors that can limit adequate energy and protein intake should be actively investigated, particularly loss of appetite that can persist for months after ICU discharge [115]. Loss of appetite and reduced oral intake can be more pronounced when reported by the patient than when objectively assessed with a standard buffet meal [118] and can be associated with severe depression [119], again indicating the need for a multidisciplinary approach in these patients. No specific guidelines on macro- and micronutrient requirements are available for ICU survivors, but experts suggest that nutritional requirements are higher than in healthy patients [120] and may remain so for long [121]. Oral nutrition supplements with high-protein and β-hydroxy β-methyl butyrate may be useful after post-hospital discharge. Propranolol, oxandrolone, and other anabolic/anti-catabolic agents may also help to restore lean muscle mass [121] and might be incorporated into a patient-centered nutritional approach with regular dietician follow-up visits.

## Conclusions and future directions

Severe weakness is a common complication of critical illness impacting short and long-term outcomes and should be actively sought in all hospitalized patients with serious illnesses. No effective pharmacological treatments are available, but early ICU mobilization adapted to the specific patient’s needs together with the best evidence-based ICU practices, such as the ABCDEF bundle, can effectively improve muscle strength.

Weak patients seen at follow-up need appropriate multi-dimensional evaluation and close multi-professional collaborations. Follow-up clinics may serve as a hub to connect and coordinate different medical specialists, physiotherapists, dieticians, psychologists, and family physicians into a unitary physical, cognitive, and psychosocial community care program for survivors of critical illnesses.

Future studies should assess whether a comprehensive approach with pharmacological treatments, and rehabilitation and nutritional strategies begun during hospitalization and maintained for a sufficiently long time after hospital discharge combined with adequately planned post-ICU and post-hospital care transitions can effectively reduce CIW.

## Data Availability

Not applicable.
