# Publisher Correction to: Critical Illness Weakness, Polyneuropathy and Myopathy: Diagnosis, treatment, and long‑term outcomes

**DOI:** 10.1186/s13054-023-04757-3

**Published:** 2023-11-30

**Authors:** Nicola Latronico, Frank A. Rasulo, Matthias Eikermann, Simone Piva

**Affiliations:** 1 Department of Medical and Surgical Specialties, Radiological Sciences and Public Health, University of Brescia, Brescia, Italy; 2 Department of Emergency, ASST Spedali Civili University Hospital, Piazzale Ospedali Civili, 1, 25123 Brescia, Italy; 3 “Alessandra Bono” Interdepartmental University Research Center on Long‑Term Outcome (LOTO) in Critical Illness Survivors, University of Brescia, Brescia, Italy; 4 Department of Anesthesiology, Montefiore Medical Center, Albert Einstein College of Medicine, Bronx, NY USA

**Publisher Correction : Critical Care 27, 439 (2023) ** **https://doi.org/10.1186/s13054-023-04676-3**

Following publication of the original article [1], an incomplete title was published. The word ‘Critical’ was missing from the title.

The incorrect title is: Illness Weakness, Polyneuropathy and Myopathy: Diagnosis, treatment, and long-term outcomes

The correct title is: Critical Illness Weakness, Polyneuropathy and Myopathy: Diagnosis, treatment, and long‑term outcomes

The publisher sincerely apologize to the authors and readers for the inconvenience caused.

The title has been updated in this publisher correction and the original article [1] has been corrected.
